# Recent advances in the application of microbiota transplantation in chickens

**DOI:** 10.1186/s40104-025-01233-6

**Published:** 2025-06-27

**Authors:** Haoran Zhao, Luke Comer, Muhammad Zeeshan Akram, Matthias Corion, Yang Li, Nadia Everaert

**Affiliations:** 1 Nutrition and Animal-Microbiota Ecosystems Laboratory, Department of Biosystems, KU Leuven, Heverlee, Belgium; 2 Key Laboratory of Efficient Utilization of Non-Grain Feed Resources, Ministry of Agriculture and Rural Affairs, College of Animal Science and Technology, Shandong Agricultural University, Panhe Street 7, Tai’an, 271017 China

**Keywords:** Behavior, Gut health, Immunity, Metabolism, Microbiota colonization, Microbiota composition, Microbiota modulation

## Abstract

Extensive evidence demonstrates that a healthy and well-balanced gut microbiota profoundly influences host nutrient absorption, immunity, and metabolism. Unlike mammals, early microbiota colonization in commercial poultry largely depends on the environment as chicks hatch in incubators under a relatively sterile environment (egg and incubator sterilization) without maternal-offspring interaction. The early gut microbiota remains unsaturated, providing a critical window for modulation and influencing the subsequent microbiota succession, which may have long-term health outcomes. Microbiota transplantation (MT) involves transferring the microbiota from a donor to a recipient to modulate the recipient’s microbiota toward a desired state. Successfully applied in human medicine, MT is also gaining attention in poultry production to modulate intestinal health. This review comprehensively explores factors affecting MT, its mechanisms, and its potential applications in chickens, providing insights for further research and commercial use.

## Introduction

The commercial chicken differs from other farm animals in the establishment of its microbiota, largely due to unique management practices. Eggs are typically cleaned or fumigated before hatching, there is no contact with adult chickens during incubation, and no maternally-provided interactions post-hatch [1]. Although some studies suggest that a limited number of microbes may colonize the inside of the egg before hatching via vertical transmission from the hen, this number is minimal due to the lack of contact with the parents [2, 3]. The initial composition of the chick’s gut microbiota is primarily derived from the first bacterial colonizers obtained from the chick’s environment, which has been reported to influence microbiota succession and host health [4, 5]. Indeed, the chick gut microbiota is more likely to be acquired from the hatchery, post-hatching transportation, or directly from the chicken house itself. This lack of early exposure to a range of health-promoting microorganisms increases the risk of pathogen colonization, as harmful bacteria are widely distributed in the chicken house, feed, and drinking water [5]. The early colonizers, influenced by dietary and management-related factors, play a critical role in shaping the subsequent composition of the gut microbiota [4–6]. This early life stage therefore represents a crucial period for gut microbiota establishment in chicks [7]. Proper acquisition and maturation of the gut microbiota are essential for developing and maintaining gut immune function, barrier integrity, and nutrient metabolism, all of which directly impact the growth performance and overall health in chickens [8].

Microbiota transplantation (MT) has emerged as an effective strategy for shaping the gut microbiota by transferring a donor’s microbiota to the recipient to establish a more favorable microbial composition [9]. The successful clinical application of MT in treating *Clostridioides difficile* infections in humans demonstrates its effectiveness in disease prevention and treatment by modulating the microbiota [10]. In humans, MT has also been used to alleviate inflammatory bowel disease and metabolic disorders [11, 12]. MT has been extended to livestock species where it has been implemented with promising results. For instance, piglets recovered from necrotizing enterocolitis after receiving a microbiota inoculum which was shown to facilitate a healthy microbial ecosystem [13, 14]. In poultry, growing evidence suggests that MT supports the establishment of gut microbiota without disruption, inducing significant phenotypic changes and improving health and performance metrics. MT in day-old broilers for instance ameliorated necrotic enteritis by modulating the intestinal microbiota and host immune responses [15]. With an increasing focus on the potential of MT to directly regulate chicken gut microbiota, this review aims to explore its applications in poultry production. Key topics include the history of MT, donor selection, delivery methods, mechanisms of action, alongside specific effects on behavior, production and immunity, providing a reference for the application of MT in poultry health and production.

## Composition and early microbiota development

### The microbiota along the gastrointestinal tract

This section focuses on the core microbiota of adult chickens, which exhibits distinct regional characteristics throughout the gastrointestinal tract (GIT). For a detailed discussion on age-related changes in gut microbiota, please refer to Dai et al. [16]. The microbiota of the chicken GIT is highly diverse, comprising over 900 bacterial species [17]. In the crop, *Lactobacillus *(*L.*) species predominate, playing a critical role in starch fermentation and lactate production [18]. Among these, *L. reuteri*, *L. johnsonii*, *L. crispatus*, *L. gallinarum*, and *L. amylovorus* are consistently detected, despite age-related shifts in *Lactobacillus* population [19, 20]. The proventriculus and gizzard, characterized by low pH, serve as an essential filtration barrier to prevent bacteria from entering the hindgut [20]. *Lactobacillus* species also dominate these regions, likely due to their ability to thrive in acidic environments [18]. Additionally, *Enterococcus* species and lactose-negative *Enterobacteria* are also frequently present [21]. Although the bacterial concentration in the proventriculus and gizzard is comparable to that of the crop, microbial fermentation activity there is relatively low [20]. Throughout the length of the small intestine, microbial density varies considerably. The proximal small intestine has a lower bacterial concentration, primarily due to the short transit time of feed and the presence of abundant digestive enzymes, pancreatic secretions and bile acids [20]. In contrast, the distal small intestine has reduced digestive enzyme activity and deconjugated bile acids, creating a more favorable environment for bacterial growth [20]. Despite these differences in concentration, the microbial composition across the small intestine is relatively consistent, with *Lactobacillus* remaining a key bacterial species [4, 18].

The cecum, a primary site for fermentation, exhibits the highest taxonomic abundance and microbial diversity. The cecal microbiota plays a critical role in breaking down cellulose, starch, and polysaccharides that escape digestion in the small intestine [18, 22]. The phyla Firmicutes, Bacteroidetes, Actinobacteria, and Proteobacteria are detected in the ceca of nearly all adult chickens. In healthy adult hens, Firmicutes and Bacteroidetes are typically balanced, each accounting for approximately 45% of the total microbiota. Meanwhile, Actinobacteria and Proteobacteria are less abundant, representing only 2%–3% of the total microbial community [4, 18]. However, these proportions are not absolute, as significant individual variability exists in chickens’ cecal microbiota. Studies have reported that the proportion of Bacteroidetes can range from 10% to 90% without indicating abnormal behavior [18]. Similarly, the relative abundance of Actinobacteria and Proteobacteria has been found to exceed 10% in some cases [18].

The colonic microbiota is influenced by the unique flow of the chyme in the GIT of chickens. Specifically, given the short length of the colon, most chyme passes directly from the small intestine to the colon, while a smaller portion enters the cecum for fermentation before being released into the colon [18, 23, 24]. As a result of this dynamic flow, the colon's microbiota may resemble that of the ileum or the cecum, or a combination of the two [18].

### Early microbiota colonization

Gut microbiota colonization in chickens begins during the embryonic stage, although the microbial load is initially limited [2, 3, 25]. The poultry reproductive tract, which has a relatively high abundance of *Lactobacillus*, serves as the site for the formation of various egg components, including the yolk, albumen, eggshell membrane, and eggshell [26]. Bacteria may be directly deposited onto these components during oogenesis, prior to oviposition [16]. The yolk, providing over 90% of the nutrients needed for embryonic development, serves as an important nutrient reservoir [3]. Contrary to prior assumption, recent studies [3] indicate that the yolk may not be sterile, and the microorganisms inhabiting this site are functionally associated with amino acid, carbohydrate, and lipid metabolism during chicken embryogenesis [3]. During embryonic development, the relative abundance of Firmicutes and Bacteroidetes in the yolk increases from d 7 to 15 but decreases from d 15 to 19 [3].

Additionally, some studies indicate that chicken embryos acquire a gut microbiota from the egg albumen before hatching, although the mechanism by which microbes bypass the antimicrobial properties of albumen remains unclear [2]. From d 3 to 12 of embryonic development, microbial diversity increases in the gut, which may correlate with the formation of different organs [25]. However, diversity declines sharply by d 19, with some species disappearing altogether [25]. This phenomenon may be attributed to two factors: firstly, the maturing immune system becomes increasingly complex and aggressive, restricting microbes to extracellular spaces; secondly, facultative anaerobes, which dominate during early embryonic development, are gradually replaced by obligate anaerobes, explaining the disappearance of certain microbes [25].

The cloaca, the final anatomical site the egg passes through before being laid, plays a significant role in microbial colonization. Cloacal bacteria colonize the eggshell surface before exposure to environmental bacteria [2]. Anatomically, the cloaca connects to the terminal ends of the digestive and urinary systems, meaning the eggshell may also be contaminated by feces [26]. Microbiota on the eggshell surface significantly contribute to microbial colonization, as research demonstrates that sterilized eggshells result in a distinct gut microbiota in hatched chicks compared to untreated eggshells [27]. Additionally, the hatching environment significantly impacts the gut microbiota of chicks [28]. This influence is further highlighted by comparisons of the gut microbiota on d 19 of incubation and immediately after hatching, which reveal a notable increase in the abundance of Firmicutes following hatching [3].

In commercial chicken production, hatching eggs are removed from any maternal influences, and are incubated in a sanitized environment, resulting in abnormal and random microbiota colonization with significant variability between batches [1]. Upon hatching, the few initial colonizers of the gut gain a competitive advantage, attaching to epithelial cells without competition, rapidly establishing themselves, proliferating, and shaping the gut environment to suit their own requirements [29]. *Escherichia coli* (*E. coli*), a widely distributed facultative anaerobe, survives easily in the environment and is highly likely to come into contact with newly hatched chicks. As a result, *E. coli* is typically one of the first colonizers of the gut microbiota in commercially hatched chickens, dominating the cecum during the first week post-hatch [18, 26]. Microbes’ presence in feed can also have a significant impact on colonization. Research indicates that *Lactobacillus* are observed in the crop and cecum as early as 4 h after providing chicks with feed, with colonization extending to the duodenum and ileum within 24 h [30].

Hence, the maternal microbiota is only vertically transmitted to chicks to a limited extent, while the microbiota of chicks is more likely influenced by environmental factors. These microbes may settle on the outer surface of the eggshell, and the incubator environment often harbors abundant external bacteria, which are more likely to originate from humans rather than poultry. Without intervention, the pioneer colonizers in commercial chicks can frequently consist of pathogens derived from the eggshell exterior and the hatchery environment [6]. Early microbial intervention has a profound impact on the host. Studies have shown that microbial exposure from the eggshell significantly influences the gut microbiota composition of chickens at six weeks of age [27]. The development of the gut immune system parallels the development of the gut microbiota [16]. The immune cells of newly hatched chicks rapidly develop and mature within the first two weeks, and the dominant microbiota during this stage are closely related to immune cell development [31]. Broilers grow quickly with a short growth cycle, usually 35 to 42 d, and the effects of early exposure can persist until 35 d post-hatch, thus demonstrating the importance of microbial exposure in the first two weeks of a broiler’s life [22]. Strategically modulating the gut microbiota during this critical period holds great potential for improving the health and production performance of commercial chickens.

## Microbiota transplantation

Fecal microbiota transplantation (FMT) involves the transfer of a donor’s microbiota to a recipient in order to establish a more desirable microbiota composition [9]. The earliest documented record of FMT dates back to the fourth century in China, where it was described in the *Handbook of Prescriptions for Emergencies* [32]. This manual reported the use of "yellow soup" to treat patients suffering from food poisoning or severe diarrhea. Notably, for aesthetic reasons, the term "fecal suspension" was avoided, and instead, the term "yellow soup" was used [32, 33]. Subsequently, in the sixteenth century, the Italian physician Hieronymus Fabricius ab Aquapendente conceptualized FMT by introducing the term "transfaunation", which refers to the transfer of gastrointestinal content from a healthy donor to a diseased animal. This practice was later widely adopted in veterinary medicine [33]. It was not until the 1950 s that FMT was first reported in Western human medicine, where physicians successfully treated four critically ill patients with pseudomembranous colitis, often caused by *Clostridioides difficile* infection (CDI), using fecal material from healthy donors [34]. In 2013, the first randomized controlled clinical trial further confirmed the efficacy of FMT in treating CDI [35], with current success rates exceeding 90% [33]. Today, the application of FMT in humans has gradually expanded to include research on its potential to treat extra-intestinal diseases [33].

**Fig. 1:**
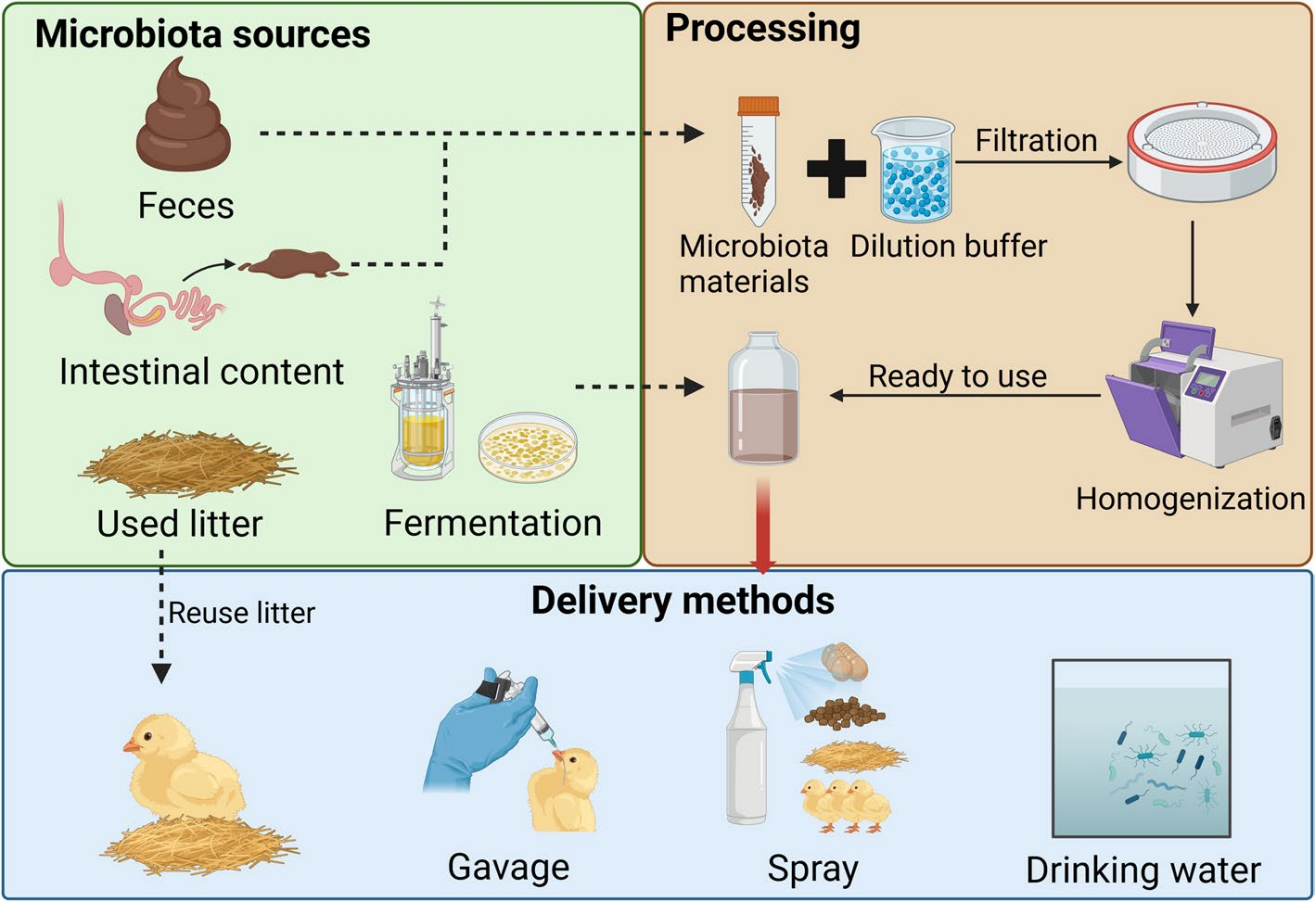
Overview of microbiota sources, processing steps, and delivery methods of microbiota transplantation (MT). Microbiota sources for MT include feces, intestinal content, fermentation products, and used litter. Feces and intestinal content are typically diluted with a buffer (e.g., saline or phosphate-buffered saline), homogenized, and filtered to remove large particles, after which they are ready for use. Fermentation products are generated by culturing healthy donor microbiota in nutrient-rich bioreactors for several days, resulting in a ready-to-use product. Delivery methods vary, with gavage being the most common approach. The prepared inoculum can also be sprayed onto eggs, feed, wood shavings, or directly onto chicks. Additionally, microbiota can be delivered through water. Reusing litter directly is also suggested, as it contains mature and abundant microbiota. Figure created in https://BioRender.com

Research and the application of FMT in poultry are still in the developmental stage. In poultry production, the source of the microbiota for transplantation is not limited to feces. Instead, it can be derived from intestinal chyme or the fermented products of intestinal contents. Therefore, the generalized term ‘microbiota transplantation’ (MT) is favored in this context to encompass all possible microbiota sources. In the following sections, we will explore the key aspects of MT and the necessary practical considerations for chickens. Firstly, we will explore the sources of MT, which can be broadly categorized into feces, intestinal content, and other potential sources (Fig. 1). Next, we will discuss the criteria for donor selection. Finally, we will discuss the methods of administering MT (Fig. 1).

### Microbiota sources

The fecal microbiota is one of the most commonly used inocula in MT due to its ability to reflect the gut microbial composition of the donor. Additionally, it is a less invasive option, minimizing the need for animal slaughter and the associated risks of invasive sampling from the intestinal tract. However, fecal samples are often exposed to oxygen, causing uncontrolled inactivation of some anaerobic bacteria [36]. Although certain anaerobes like *Anaerostipes*, *Anaerotruncus*, *Bacillus*, *Blautia*, *Dorea*, *Pseudoflavonifractor* and *Subdoligranulum* form spores and colonize the recipient’s gastrointestinal tract, oxygen exposure remains a concern [37]. Chicken feces mix with urine from the urinary system in the cloaca before being excreted [26, 38]. As a result, feces typically contain uric acid, which appears as a white, paste-like substance [38]. While most researchers take this into account when collecting fecal samples, some do not. Uric acid is the primary end product of nitrogen metabolism in chickens and may adversely affect recipient metabolism if included in the inoculum for MT [39]. The microbiota sources derived from feces are presented in Table 1.

**Table 1:** Characteristics of donors, recipients, and microbiota transplantation parameters from fecal sources

	Donor	Donor traits	Recipient	Dosage	Time	Ratio	Focus	References
1	Cobb 500, D 30	Low residual feed intake	Newly hatched Cobb 500 broiler	Gavage with 0.1 mL	Upon arrival, D 6, D 9	1:2 (w:v), 10^4^ CFU	Performance	[40, 41]
2	Turpan cockfighting × White Leghorn chickens, 3 months old	High body weight	Newly hatched yellow feather chick	Gavage with 1 mL	28 d	1:6 (w:v)	Immune	[42]
3	Cobb 500, same age with recipient	High feed efficient	Newly hatched Jing Hong laying strain	Gavage with 1 mL	D 1 to D 28	1:6 (w:v)	Performance	[43]
4	Hyline brown, peak egg production period	High and low egg production	Hens with high and low egg production	Gavage with 2 mL	Twice per day, 3 weeks	1:5 (w:v)	Performance	[44]
5	Broilers, 52 weeks	More positive physiological functions and behaviors	Newly hatched commercial broiler	Gavage with 0.5 ml	From D 5 to D 12	1:5 (w:v)	Performance, behavior	[45]
6	Broilers, 5 weeks of age	*Campylobacter*-free	*Campylobacter* infection model	Gavage with 0.1 mL	D 0	1:20 (w:v)	Immune, pathogen resistance	[46]
7	Arbor Acres broiler, Beijing-You broiler	Difference in meat quality	Newly hatched Arbor Acres broiler	Gavage with 1 ml	D 0 to D 21	1:1 (w:v)	Meat quality	[47]
8	Turpan cockfighting × White Leghorn chickens, 7 weeks	High body weight	Newly hatched Turpan cockfighting × White Leghorn chicken	Gavage with 1 mL	30 d	1:6 (w:v)	Performance, immune	[48]
9	Wumeng black-bone chickens, D 42	Circadian misalignment	Wumeng black-bone chickens, D 42	Gavage with 0.2 mL	7 d	1:5 (w:v)	Circadian misalignment	[49]
10	Hyline brown, 6 months	Healthy	Newly hatched Hyline brown	Drinking water	6 d	1:8 (w:v)	Immune	[50]
11	Hyline brown breeder, 6 months	Healthy	Newly hatched laying-type	Gavage with 0.1 mL	D 1, D 3, and D 5	1:2 (w:v)	Immune, gut	[51]
12	Parent breeders, 55 weeks	High egg production	Microbiota-depleted breeders, 55 weeks	Gavage with 2 mL	4 weeks	1:5 (w:v), 1.4 × 10^12^ CFU/mL	Ovarian function	[52]
13	Hyline brown and Jilin Yellow hens, 5 to 6 months	Autologous and heterologous	Newly hatched Hyline brown	Gavage with 0.1 mL or through dring water	D 1, D 3, and D 5 for gavaging; through drinking water for 6 d	1:2 (w:v) for gavaging; 1:8 (w:v) for drinking water	Performance, immune, intestinal	[53]
14	Leghorn chicken × Turpan fighting chickens, 12 months	High and low abdominal fat	Newly hatched white feather broiler	Gavage with 1 mL	28 d	1:6 (w:v)	Fat metabolism	[54]
15	White Leghorn layers	Parental microbiota	Eggs from white Leghorn layer	Feces on the egg	NA	NA	Microbiota	[27]
16	SPF white Leghorn chickens, D 28	*Mycoplasma gallisepticum* infection	SPF white Leghorn chickens, 42 d	Gavage with 0.5 mL	7 d	1:5 (w:v)	Immune, infection	[55]
17	Three-yellow chickens, D 42	Immunization with *Eimeria tenella*	Newly hatched three-yellow chick	Through the cloaca or gavage	D 1 and D 7	10, 5, and 1 g of fecal bacteria/mL	Immune, pathogen resistance	[56]
18	Arbor Acres broiler, D 35	High and low body weight	Newly hatched Arbor Acres broiler	Gavage with 0.2 mL	14 d	1:2 (w:v), 10^9^ CFU/mL	Antioxidant capacity, gut, immunity	[57]
19	Female chickens, D 70	Low residual feed intake	Newly hatched chick from high residual feed intake line	Gavage with 0.2 mL	35 d	1:6 (w:v)	Performance	[58]

*D* Days, *NA* Not available, *SPF* Specific pathogen free, *CFU* Colony-forming units

Some studies have focused on obtaining the microbiota directly from the gastrointestinal tract, as microbiota from intestinal chyme more accurately reflects the donor's gut microbiota compared to fecal samples (Table 2). For instance, van der Eijk et al. [59] and Li et al. [60] collected whole intestinal chyme from the gastrointestinal tract, pooled to create an inoculum, which was then transplanted into chicks. Similarly, Liu et al. [31] collected ileal samples from broilers at 14- and 35-days post-hatch. In addition, cecal content is frequently used due to the cecum’s role as the primary sites of microbial fermentation and its high microbial concentration. However, collecting intestinal content is challenging, as it typically requires sacrificing the animal to obtain the material through dissection.

**Table 2:** Characteristics of donors, recipients, and microbiota transplantation parameters from intestinal content

	Material	Donor	Donor traits	Recipient	Dosage	Time	Ratio	Focus	References
1	Cecal content	White Leghorn SPF, D 208	Healthy	Newly hatched White Leghorn SPF	Gavage with 0.1 mL	D 0	1:10 (w:v)	Immune, pathogen resistance	[61]
2	Cecal content	6_3_ and 7_2_ lines	Resistance or susceptibility to Marek’s disease	Newly hatched male Dekalb XL strain	Gavage with 0.1 mL	D 1 to D 10, weekly from week 3 to week 5	1:10 (w:v)	Behavior, immune	[62–64]
3	Cecal content	Arbor Acre broiler, D28	Healthy	Arbor Acre broiler, D 5	Gavage with 0.2 mL	D 5 to D 10	1:10 (w:v)	Intestinal health	[65]
4	Cecal content	Qingyuan partridge chickens, D 140	High abdominal fat	Newly hatched Qingyuan partridge	Gavage with 1 mL	D 0 to D 9	1:100 (w:v)	Fat metabolism	[66]
5	Cecal content	Roslin broilers, 40-week	Different breed with recipient	Newly hatched Ross 308	Gavage with 0.1 mL	D 0	1:6 (w:v)	Microbiota	[67]
6	Cecal content	Arbor Acres broiler, D 14, D 28	Diet supplied with folic acid	Arbor Acres broiler, D 14	Gavage with 1 mL	D 14 to D 28	NA	Fat metabolism	[68]
7	Cecal content	Broilers, 2 and 6 weeks	Cross generation	Newly hatched chicks	Gavage with 0.2 mL	D 0	3:1 (w:v)	Immune, pathogen resistance	[69]
8	Cecal content	6_1_ and N lines, 3 weeks	Resistance to *Campylobacter jejuni*	Homologous and heterologous transplants	Gavage with 0.1 mL	D 0	1:6 (w:v)	Immune, pathogen resistance	[70]
9	Cecal content	Turpan cockfighting × White Leghorn chickens, 7 weeks	High body weight	Newly hatched Turpan cockfighting × White Leghorn chickens	Gavage with 1 mL	30 d	1:6 (w:v)	Fat metabolism	[71]
10	Cecal content	Arbor Acre broiler, D 28	Healthy	Arbor Acre broiler, D 8	Gavage with 0.3 mL	At D 8, 3 consecutive days	1:10 (w:v)	Immune	[72]
11	Cecal content	Brown Lohmann laying hens, 86-week-old; broiler, 6 weeks	Different breeds	Cobb broiler eggs	Brushing 50 mL on eggs, sprayed 100 mL on wood shaving, Gavage with 0.2 mL to chicks,	Once	1:25 (w:v)	Immune	[73]
12	Cecal content	Ross 308, Hubbard JA87, and Cobb 500, D 42	Different breeds	Ross 308 eggs	Eggs were sprayed with 5 mL of diluted cecal content	D 2, D 7, D 14, D 18 of incubation	1:20 (w:v)	Microbiota	[74]
13	Cecal content	NA	Different ages	SPF hens, D 12	Gavage with 1 mL	D 12 to D 15	4:5 (w:v), 10^9^ bacterial cells	Immune	[75]
14	Cecal content	Arbor Acre broiler, D 42	Healthy	Newly hatched Arbor Acre	Gavage with 0.05 mL	D 0 and D 1	1:10 (w:v)	Microbiota	[76]
15	Cecal content	ISA Brown, different ages	Different ages	Newly hatched SPF ISA Brown chickens	Gavage with 0.1 mL	D 0	1:10 (w:v)	Microbiota, protein metabolism	[77]
16	Cecal content	Cobb 500 broiler, D 25	High and low feed conversion ratio	Cobb 500 fertile eggs	Painted onto the eggs, 0.3 mL	On the day before hatching	1:1 (w:v)	Microbiota	[78]
17	Cecal content	Wannan Yellow Chicken, 80 weeks	Healthy, pathogen free birds	Newly hatched Wannan Yellow chickens	Spray to feed, 1 mL/20 g feed	60 d	1:12 (w:v) and 1:6 (w:v)	Performance	[79]
18	Cecal content	ISA Brown, different ages	Different ages	Newly hatched ISA brown	Gavage with 0.2 mL	D 0	1:5 (w:v)	Microbiota	[37]
19	Content of the ileum, cecum and colon	White Leghorn, 10 and 30 weeks	High and low feather pecking	Different feather pecking traits	Gavage with 0.1 mL	14 d	2:1 (w:v)	Behavior	[59]
20	Ileal content	Arbor Acres male chicken, D 14, D 35	Different ages	Newly hatched Arbor Acres	Gavage with 0.2 mL	D 0 and D 3	10^9^ CFU	Immune, pathogen	[31]
21	Whole intestinal content	Broiler, D 21	Commerical broilers (with bacteria)	Bacteria free chicken, D 21	Gavage with 1 mL	D 21 to D 28	1:2 (w:v), 10^8^ CFU/mL	Microbiota function	[60]

*D* Days, *NA* Not available, *SPF* Specific pathogen free, *CFU* Colony-forming units

To address this, Gong et al. [80, 81] developed a chemostat fermentation method, where broiler cecal samples were fermented over 11 d. The resulting inoculum was then administered to chicks. Similarly, Zaytsoff et al. [15] used a continuous-flow bioreactor system that combined broiler breeder cecal samples with nutrients such as proteins and vitamins. After 10 days of fermentation, the prepared material was used in their study. Another approach used cultured cecal microbiota grown on brain–heart infusion plates, and transferred to newly hatched chicks to combat *Campylobacter jejuni* infections [82]. Additionally, used litter from chicken flocks has also been explored as a microbiota source for MT due to its mature, rich, and diverse microbial community. In regions like the United States and Brazil, reusing litter across multiple broiler flocks is common practice [83]. In contrast, Canada and Europe recommend fresh litter for each flock to minimize the risk of pathogen transmission [83]. Ramírez et al. [69] investigated the effects of microbiota obtained from built-up litter on *Salmonella enterica* and *Campylobacter jejuni* resistance. Furthermore, Oladeinde et al. [83] evaluated the application of reused litter from commercial poultry farms to combat *Salmonella* Heidelberg. Despite these developments, research on the efficacy and safety of litter as a microbiota source remains limited, hence requiring further investigation. Microbiota sources other than the feces and intestinal content which can be used for MT in chicken are presented in Table 3.

**Table 3:** Characteristics of donors, recipients, and microbiota transplantation parameters from other sources

	Material	Donor	Donor traits	Recipient	Dosage	Time	Ratio	focus	References
1	Fermentation	Broiler breeder, 6 months	Healthy	Newly hatched Ross 308 FF	Gavage with 1 mL	once	Fermentation	Immune, pathogen resistance	[15]
2	Fermentation	Broiler, D 180, ferment cecal content for 11 d	NA	Newly hatched broiler chicks	Gavage with 0.5 mL	once	Fermentation 10%	Microbiota	[81]
3	Fermentation	Lohmann white layer, 40 weeks; Bantam rooster, 40 weeks; Cornish Cross Rock broiler, D35	Different genetic lines and ages	Newly hatched Ross 708	gavage with 0.15 mL/spray technique, 2 mL	once	Fermentation	Microbiota, intestinal health	[84]
4	Fermentation product	Fermented, undefined cultures from intestinal microbiota of chickens	Healthy SPF chicken	Newly hatched Ross 308	Gavage with 0.5 mL	once	Fermentation product	Immune	[85]
5	Fermentation	Cobb broiler, D 28	Diet supplied with secondary bile acid	Newly hatched Cobb broilers	NA	once	Fermentation, 10^8^ CFU/bird	Immune, pathogen resistance	[82]
6	Fermentation	Broiler, D 180, ferment cecal content for 11 d	NA	Newly hatched broiler chicks	Gavage with 0.5 mL	once	Fermentation 10%	Intestine health	[80]
7	Feces litter	Broiler chicken	Simulated commercial farm conditions	Newly hatched Cobb 500	Reused litter topped up with 0.5 cm of fresh pine shavings	14 d	NA	Immune	[83]
8	Built-up litter	NA	Environmental microbiome	Newly hatched chicks	Gavage with 0.3 mL	once	3:1 (w:v)	Immune	[69]

*D* Days, *NA* Not available, *SPF* Specific pathogen free, *CFU* Colony-forming units

### Donor selection

In the application of MT in poultry, the selection of donors is guided by various criteria. Age is a critical factor, as the composition of the microbiota evolves over time, with mature donors often providing a more stable and diverse microbial community [16]. In contrast, younger donors may introduce instability due to their less-established microbiota [18]. For instance, Volf et al. [77] and Ondrej et al. [37] found that recipient responses varied with donor age when microbiota from donors aged 1, 3, 4, 16, 28, 40, and 42 weeks were transplanted, with notable differences in the expression of more than 250 cecal tissue proteins, among which immune related protein, fibrinogen-like domain proteins, and cysteine-rich scavenger domain-containing proteins were the most prominent.

The ideal outcome of MT is to transfer desirable traits from donors to recipients, which is why specific breeds are often selected for their unique characteristics. For example, breed-specific differences have been explored using chickens from lines 6_3_ and 7_2_, known for their differing resistance to Marek’s disease [62–64], or lines 6_1_ and N, chosen for their varying resistance to *Campylobacter jejuni* [70]. Similarly, Song et al. [50, 53] utilized Hy-Line Brown hens (high egg production) and Jilin Yellow chickens (disease-resistant) as donors to enhance the immune function and intestinal health of newborn laying chicks. Lei et al. [47] selected Arbor Acres and Beijing-You broilers, known for their distinct meat qualities, to investigate whether transferring their microbiota could influence meat characteristics. Additionally, the Qingyuan breed and Turpan cockfighting × White Leghorn hybrids have garnered attention for their unique fat metabolism traits [54, 66, 71]. Similarly, Richards-Rios et al. [74] and Feng et al. [65] selected different breeds, including Ross 308, Hubbard JA87, and Cobb 500 as cecal donors based on distinct microbiota maturation patterns. Studies have also explored breeds such as the Wannan Yellow Chicken [79], Lohmann white layer [73, 84], and Roslin broilers [67].

Chickens are important economic animals, and their growth performance is a key area of focus in broiler production. Donors with superior traits can transfer microbiota that may enhance these characteristics in recipients, making MT a promising strategy for improving productivity. For instance, Siegerstetter et al. [40], Metzler-Zebeli et al. [41], Xie et al. [58], and Donaldson et al. [78] selected high-feed-efficiency broilers as donors. Similarly, Elokil et al. [43] extended this approach by selecting highly feed-efficient broilers as donors to improve body weight in laying hens. Body weight, a crucial indicator of chicken growth and health, has been a focus of several studies. Chickens with different body weights from 3 different breeds were selected as donors [42]. Zhang et al. [48, 57] conducted similar research using chickens of varying body weights within the same batch. Egg production, another key metric in the poultry industry, has been targeted with fecal microbiota transfer. Cao et al. [52] and Wang et al. [44] selected donors from high- and low-egg-production hens and observed changes in egg production performance and ovarian function in recipients.

Donors selected based on robust immune responses aiming to improve disease resistance in recipients by suppressing harmful pathogens, can contribute to improved gut health and reduced disease susceptibility in the recipient population. For instance, Pang et al. [46] used non-*Campylobacter*-colonized donors to enhance resistance to *Campylobacter* in recipients. Pottenger et al. [86] used cecal microbiota from eight-week-old Ross 308 birds to protect newborn chicks from *Salmonella* Typhimurium. Similarly, Zaytsoff et al. [15] used a cecal microbiota from six-month-old broiler breeders to mitigate the side effects of necrotic enteritis in chicks. Yitbarek et al. [75] used age-matched donors to assess immune responses to the avian influenza virus H9N2. Additionally, cecal content from 28-day-old Arbor Acres chickens was used to mitigate lipopolysaccharide-induced intestinal inflammation [72]. The cecal microbiota has also shown efficacy in combating *Campylobacter jejuni* infection in chickens [69]. MT has also been used to assess the potential transmission risk of avian influenza virus from wild birds to domestic poultry, whereby heterogeneous feces from wild ducks was transplanted to laying hens during peak egg production [87].

In addition, donor selection extends beyond replicating phenotypic traits of the donor. The motivation for exploring MT as an intervention for various phenotypes stems from studies showing associations between these phenotypes and changes in the gut microbiome. However, these associations do not rule out the possibility that the observed changes in microbiota composition may be secondary consequences of the phenotype, rather than primary causative factors. Therefore, MT also serves as a valuable model for exploring the interactions between the microbiota and other factors. For instance, MT has been used to study the relationship between the microbiota and circadian misalignment (a disruption of the biological clock), using chickens with this disorder as donors [49]. Wang et al. [55] used a chicken infected with *Mycoplasma gallisepticum* as donors, transferring their fecal inoculum to microbiota-deprived chickens to investigate the microbiota's role in inflammatory injury.

### Methods of administration for microbiota transfer

The delivery methods for fecal and intestinal MT in poultry vary significantly (Fig. 1). Most studies focus on oral gavage for microbiota administration to chicks, but alternative approaches have nonetheless been explored. For instance, Khalid et al. [79] prepared a cecal inoculum and sprayed it onto feed daily, while Richards-Rios et al. [74] sprayed the inoculum onto eggs four times during incubation. Donaldson et al. [78] used cotton to wipe the inoculum onto eggs a day before hatching. Franco et al. [73] combined egg painting with wood shavings spraying before placing chicks into the pen. Spraying the inoculum directly onto chicks has also been reported by Pottenger et al. [86] and Marcolla et al. [84]. Maki et al. [27] proposed leaving feces on eggs as a microbiota delivery method. Other studies have investigated the use of drinking water as a medium to supply chicks with a microbiota [50, 53, 73, 86]. Furthermore, Lei et al. [47] introduced another method involving reused litter collected from a simulated commercial poultry production system, which was then distributed to chicks.

## How microbiota transplantation reshapes the recipient’s microbiota composition

From an ecological perspective, MT can be regarded as a form of ecological invasion, encompassing four main stages: introduction, establishment, growth and persistence, and impact [88, 89]. The success of microbial colonization depends on both the properties of the introduced microbiota and host-specific factors [89].

### Microbiota introduction

Effective colonization requires the introduced microbes to be present in sufficient quantities, exhibit high viability, and possess strong tolerance traits [88, 89]. For instance, *Fusobacterium*, *Methanobrevibacter*, *Paraprevotella*, and *Rikenella* are highly sensitive to oxygen and may lose viability during the preparation of cecal inoculum, hindering their colonization in the chicken cecum [37]. In contrast, genera such as *Alistipes*, *Bacteroides*, *Barnesiella*, and *Mediterranea* demonstrate greater adaptability, sustaining their presence in chicks for at least one week after a single inoculation and serving as effective colonizers of the broiler cecum [84]. The genetic and phenotypic diversity of introduced microbial species also plays a critical role in the success of colonization, with greater genotype diversity increasing the likelihood of adaptations necessary for establishment [89]. One key advantage of MT is its introduction of a higher diversity of species compared to probiotics [89]. Probiotics are usually formulated with a single or few species, which consequently only influence certain pathways in the gut when introduced [90]. In contrast, MT can provide a balanced and diverse bacterial composition that can quickly adapt to the gut ecosystem and foster a controlled microbiome environment [89]. While many probiotic strains can survive the passage along the GIT, their efficacy often varies widely [89, 91]. This variability arises because commercial poultry probiotics often consist of non-native strains lacking the traits required for successful integration into the gut ecosystem and also depends on the composition of the resident microbiota [89, 91].

Host factors act as habitat filters, selectively favoring certain microbes most suited for colonization [89]. The first barrier faced by microbiota introduced through MT is the gastrointestinal pH, which exerts selective pressure on microbial communities. In broilers, gastrointestinal pH ranges from 3.5 to 6.6 [92], requiring introduced microbes to exhibit strong acid tolerance. Typically, Firmicutes and Actinobacteria demonstrate greater resilience to acidic conditions [93]. For instance, *Lactobacillus* species have shown notable acid tolerance and have been successfully transplanted through MT in chickens [42]. In contrast, *Bacteroides* species are more sensitive to pH values [93]. Study reported that the relative abundance of *Bacteroides* decreased by 22% in chickens treated with MT compared to the control group [45]. However, other research has shown a significant increase in *Bacteroides* abundance in MT-treated groups [61], suggesting that pH is not the sole factor limiting microbiota survival up to the hindgut. While microbiota require a suitable pH range for survival and growth, they can employ various mechanisms to cope with extreme gastrointestinal pH. Active mechanisms include direct proton uptake or the production of enzymes that consume protons [94]. Another mechanism bacteria may use is the adjustment of membrane lipid and porins composition to reduce proton influx under acid stress [94]. Additionally, the rapid transit time of the broiler digestive tract is less than 30 min to reach the small intestine, often shorter for liquids, limiting microbial exposure to acidic environments, which likely enhance the success of MT [95, 96].

After residing in the acidic gizzard, bacteria enter the small intestine, where bicarbonate secretions neutralize gastric acid, raising the pH to approximately 6.4, which is suitable for microbiota survival [92, 97]. However, the small intestine contains abundant digestive fluids, such as digestive enzymes and bile salts, whose direct effects on microbial survival remain unclear. In newly hatched chicks, the impact of digestive enzymes on MT may be limited due to lower digestive enzyme levels [98]. Indirectly, enzymes degrade dietary components, providing nutrients that promote microbial growth [99]. Bile acids in digestive fluids exert a dual effect on microbiota [100]. Certain bacteria, such as *Clostridium scindens*, modify primary bile acids to produce secondary bile acids, which inhibit the growth of many Gram-positive bacteria [100]. In the small intestine, bacterial adhesion to host cells, mucus, or feed particles is likely critical for their persistence in the gut [100]. Besides, some microbes employ adaptive strategies, such as spore formation, to withstand environmental stress [101]. Studies administering pure bacterial cultures to newly hatched chicks found no colonization of *Lachnospiraceae*, *Ruminococcaceae*, or *Erysipelotrichaceae* isolates in the cecum by d 7, possibly because these bacteria formed spores and failed to be detected [102]. However, extended observation by 17 d revealed that a single exposure enabled colonization of 10 bacterial strains, including *Lactobacillus* and *Bacteroides* [103], indicating that spore germination requires specific conditions, such as nutrient like bile acids [100, 101, 104].

Other factors, such as immune responses, further influence microbial introduction [89]. The gut microbiota and immune system exhibit bidirectional regulation [22]. Host compartmentalization, including the mucus layer, antimicrobial peptides, defensins, and secretory immunoglobulin A (sIgA), mediates resistance to microbial colonization [101]. Conversely, the maturation of the gut immune system relies on microbial induction [22]. Consequently, in newly hatched chicks, the underdeveloped immune system has limited impacts on MT efficacy.

### Microbiota establishment

The ability of microbes to establish themselves in the gut is closely linked to the concept of an "ecological niche", which encompasses a combination of variables, such as oxygen concentration and nutrient availability, collectively determining the specific position of a species within an ecosystem [88, 89, 105]. It is widely accepted that two species occupying the same niche cannot coexist over the long term [100]. Studies on germ-free mice have revealed that when two strains of *Bacteroides fragilis* were sequentially introduced, the first strain fully occupied the niche, preventing colonization by the second [100, 106]. Similarly, introducing two distinct microbial communities to mice demonstrated that the community that was already established had a long-lasting influence on the ecological success of subsequent colonizers [107]. Competition for adhesion sites is another critical factor in niche construction [89]. Certain bacteria modify the host environment to establish their niche by inducing the production of specific host-derived sugars, which act as nutrients or adhesion sites, facilitating colonization [108]. Pathogens like *Salmonella enterica*, on the other hand, can induce intestinal inflammation, and exploit inflammation-induced byproducts as specific substrates, thereby outcompeting commensal bacteria [109]. Microbes can exploit the host’s physiological state to construct their niche. For instance, chicks younger than 3 to 4 days old still absorb yolk, rich in fats and proteins, creating a niche favorable for lipid-metabolizing microbes [110]. Notably, early-life MT has been shown to influence lipid metabolism in chickens later in life, likely due to the colonization of lipid-metabolizing microbes [57].

The effectiveness of MT can be enhanced by combining it with prebiotics like inulin, which promotes the growth of beneficial microbes and helps them occupy ecological niches. Studies in chickens have demonstrated improved outcomes with MT and inulin compared to MT alone [50, 53]. Similar results were observed in rats, where combining probiotics with indigestible carbohydrates facilitated the establishment of *Bifidobacterium adolescentis* [111].

The microbial communities in different sections of the chicken gastrointestinal tract vary, reflecting the diverse nutritional and environmental requirements of microbiota [16, 17]. The environmental needs of donor microbiota must align with the target area for successful MT. The low pH in the stomach prevents significant bacterial colonization in this non-target areas [92]. The retention time in the chicken digestive tract is very short [96], and this rapid turnover rate limits microbial colonization in non-target areas of upper gastrointestinal tract. Most MT studies select microbial communities from the hindgut, with the cecum as the target area. On one hand, the cecum provides a favorable environment for microbial growth. On the other hand, feed retention in the cecum accounts for more than half of the total digestive transit time, allowing sufficient opportunity for microbial colonization [96]. Some studies set jejunum as target area, transferring jejunal microbiota from donors to recipients, where these microbes effectively function in the recipient’s jejunum [42, 48]. Overall, information on the colonization of recipient’s gut by donor microbiota remains limited, and the fate of individual donor species following MT needs to be further explored.

### Microbiota growth and impact

Once established, resource competition becomes a key mechanism for determining species coexistence [88, 89, 105]. When ecological niches are unsaturated, any unused resources allow incoming microbes to colonize more easily, thereby increasing the success of colonization [88]. For example, MT from high-weight birds to newly hatched chicks has been shown to enhance gut sugar transport capacity and immune function [57]. Conversely, when niches are saturated, resident microbes deplete nutrients to low levels, restricting pathogen expansion [89]. For instance, studies in mice have shown that the depletion of nutrients such as dietary amino acids by a resident microbiota serves as the primary mechanism for colonization resistance against the pathogen *Citrobacter rodentium* [100].

## Effects of microbiota transplantation on recipients

Nearly all of the evidence indicates that MT can directly alter the composition of recipients’ microbiota, even with a single treatment [61, 67]. Microbiota introduced through MT colonizes the recipient's gut and subsequently exerts its effects via metabolites. Short-chain fatty acids (SCFAs) are key metabolites closely associated with poultry health. SCFAs provide 5%−15% of the host's total energy requirements, serving as an important energy source [112]. Additionally, SCFAs regulate the differentiation and proliferation of intestinal cells, thereby enhancing the barrier function against pathogen invasion [113]. Furthermore, SCFAs help lower intestinal pH, creating an environment that inhibits the growth of pathogenic bacteria [112]. Studies have reported that MT significantly increases the concentrations of key SCFAs in cecal content, including acetic acid [81], propionic acid [41, 81, 84], butyric acid [81] and valeric acid [84]. Similar effects have been noted in feces, where MT increased butyric acid levels [51]. The primary source of SCFAs is the microbial fermentation of fibers. The host requires a diverse microbiota to efficiently metabolize dietary fibers, as specific fibers often require multi-step catalysis to produce SCFAs [114]. Microbial enzymes introduced through MT play a crucial role in digesting dietary fibers in the gut. Certain key species, such as *Bacteroides thetaiotaomicron*, contribute significantly to fiber metabolism. This species is predicted to produce 14 glycosyl hydrolases, more than any other sequenced bacterium [114, 115]. Studies show that MT and inulin together have a stronger impact on gut health than either one alone [50, 53]. Moreover, transferring the microbiota from chickens fed inulin-containing diets to recipient chickens alleviates kidney damage caused by aflatoxin A [116]. The underlying mechanism may involve the introduction of mature microbiota through MT, while dietary fibers selectively stimulate beneficial microbiota, inhibit harmful ones, and establish a positive feedback loop that ultimately enhances overall chicken health.

In addition, MT has been shown to significantly enrich recipients’ microbial functions related to amino acid metabolism, secondary metabolite biosynthesis, carbohydrate metabolism, energy metabolism, and the production of enzyme families, cofactors, and vitamins [43]. Furthermore, pathways such as beta-alanine metabolism and the biosynthesis of unsaturated fatty acids were notably enriched in MT-treated groups [80], highlighting the role of MT in improving host health through multiple metabolic pathways.

**Fig. 2:**
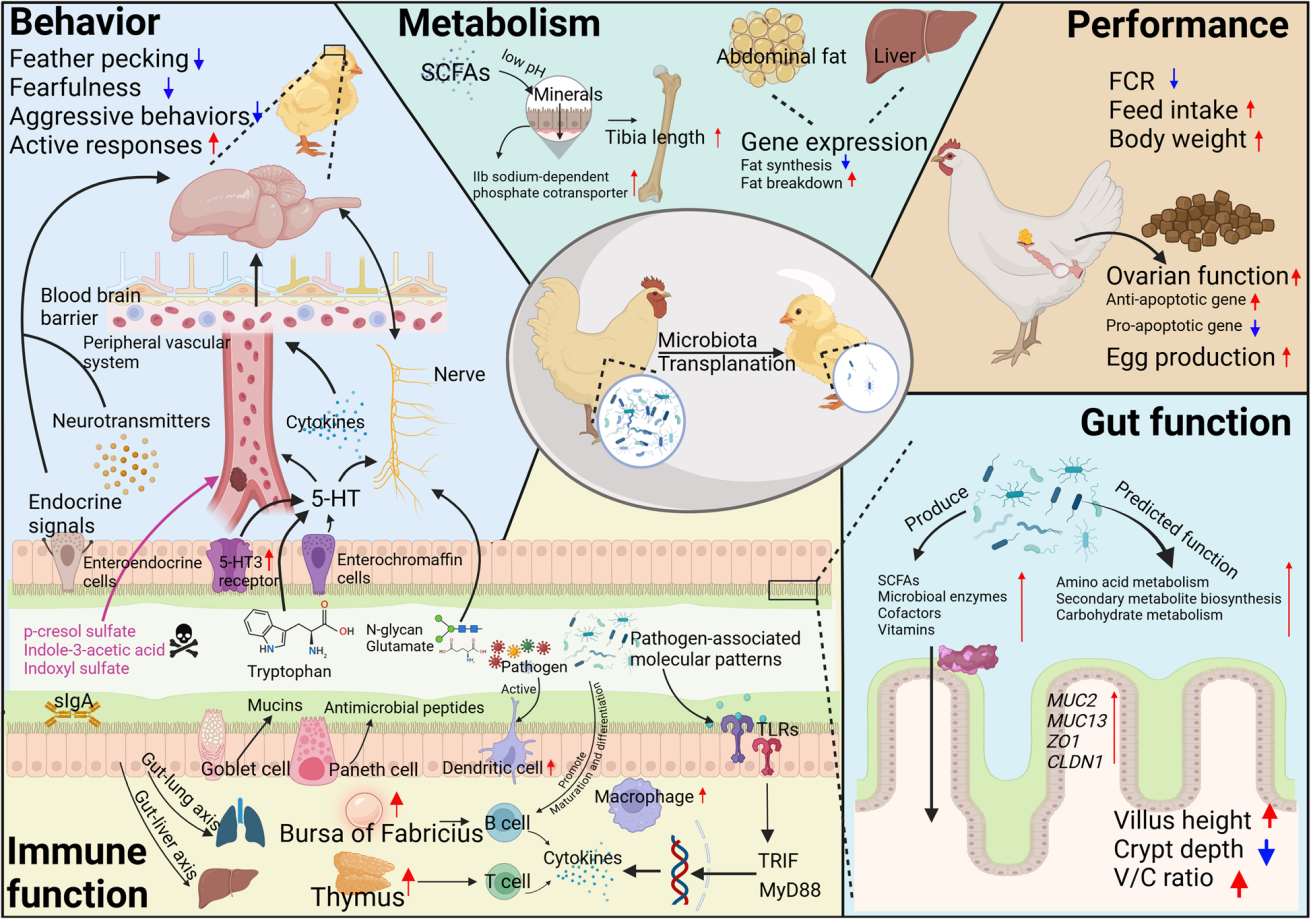
Effects of microbiota transplantation (MT) on performance, behavior, gut function, immune function, and metabolism of recipient chickens. Performance: MT enhances feed intake and body weight, reducing the feed conversion ratio (FCR). It improves ovarian function by modulating antiapoptotic and proapoptotic gene expression, hence increasing egg production. Behavior: MT influences behavior via the gut-brain axis. Microbiota-derived metabolites (e.g., p-cresol sulfate, indole-3-acetic acid) can enter the peripheral vascular system and disrupt the blood–brain barrier, facilitating cytokines to enter the brain. Serotonin (5-HT), which originates from enterochromaffin cells or luminal tryptophan, plays a key role in behavior. Additionally, the microbiota regulates behavior through endocrine signals, neurotransmitters, N-glycan, and glutamate metabolism. MT reduces feather pecking, fearfulness, and aggression while promoting active responses. Gut Function: MT-derived microbiota produce short-chain fatty acids (SCFAs), enzymes, cofactors, and vitamins, enhancing carbohydrate and amino acid metabolism, as well as secondary metabolite biosynthesis. MT improves villus height, reduces crypt depth, and increases the villus-to-crypt (V/C) ratio. It also upregulates the expression of Mucin 2 (*MUC2*), *MUC13*, Zonula occludens 1 (*ZO1*), and Claudin 1 (*CLDN1*), strengthening gut integrity. Immune Function: MT enhances gut immunity by increasing secretory IgA (sIgA), mucins, and antimicrobial peptides. Microbiota activate toll-like receptors (TLRs) via TRIF and MyD88 pathways, promoting cytokine production. MT also supports the development of the thymus and bursa of Fabricius, which are critical for T and B cell maturation, and increases dendritic cells (DCs). Additionally, MT modulates immune function through the gut-lung and gut-liver axes. Metabolism: MT improves phosphorus absorption by regulating intestinal phosphate transporters and creating an acidic gut environment via SCFAs, and also increases tibia length. MT regulates fat metabolism by modulating fat synthesis and fat breakdown genes in the liver and abdominal fat. Red upward arrows indicate promotion, while blue downward arrows indicate inhibition. Figure created in https://BioRender.com

Metabolites produced by microbiota introduced through MT have diverse effects on recipient chickens, influencing performance, behavior, gut function, immunity, and metabolism (Fig. 2). This section explores the specific impacts of MT on these aspects, highlighting its potential as a promising strategy to enhance chicken productivity and health.

### Performance

The effects of MT on growth parameters vary significantly across studies, mainly due to differences in monitoring criteria. Research on laying hens has shown that transplanting intestinal microbiota from high-producing laying hens significantly enhanced egg production rate in low-producing hens [44]. Similarly, Cao et al. [52] observed increased egg production in MT-treated layers, likely due to the improvement of ovarian function by the upregulation of antiapoptotic genes and downregulation of proapoptotic gene expression in the ovaries of the MT-treated group [52]. MT has also been shown to improve the feed conversion ratio, indicating enhanced feed utilization [43]. However, contrasting findings exist. For instance, Siegerstetter et al. [40] reported no improvement in feed efficiency when microbiota from high feed-efficient chickens were transplanted to recipients, suggesting that outcomes depend on host and environmental factors. Variability in MT effects on feed intake has also been reported. MT from the Wannan Yellow Chicken breed have a fluctuating effect on the feed intake of recipients, yet their feed intake was shown to significantly increase during the later period [79]. Moreover, donors with a low residual feed intake have been shown to transfer these traits to recipients through MT [58]. Increased body weight gain is a common outcome in recipients when receiving MT [40, 42, 43, 48, 62, 71, 79, 117], leading to a reduced time to reach slaughter weight, a result of great importance in broiler production. However, some studies have equally reported no effect of MT on body weight [45, 57].

### Behavior

Poultry in natural environments exhibit diverse social structures, with social hierarchies forming from the early stages of life and remaining relatively stable [118]. However, a number of routine management practices in modern, intensive poultry production systems may undermine the stability of social structures, adversely affecting animal production, health and welfare [119]. Poultry exhibit a range of behavioral traits, including exploratory, active, aggressive, neophobic, and fearful behaviors, showing varying degrees of heritability, depending on the specific trait being examined [120]. Interestingly, the effects of MT on these traits were independent of genetic differences between donors and recipients [64], suggesting a significant role of the gut microbiota in modulating poultry behavior.

Feather pecking (FP) is a detrimental behavior characterized by chickens pecking and pulling at the feathers of their peers, often reflecting an inability to deal with a restrictive environment [121]. FP is strongly correlated with behavioral and physiological dysfunctions [121]. Microbiota derived from high feather pecking (HFP) and low feather pecking (LFP) chicken lines can elicit more active responses in their respective lines after MT, suggesting that homologous microbiota may lower the risk of FP [59]. However, the recipients did not fully acquire the behavioral traits of the donors, highlighting the complexity of microbiota-behavior interactions [59]. Moreover, these findings offer practical implications, particularly regarding the potential of maternal microbiota interventions as a strategy to reduce FP. Additionally, the authors emphasized the importance of considering the recipient's genotype in practical applications, as MT influenced HFP recipient behavior during treatment, whereas the effects on LFP recipients emerged only after treatment [59].

Research has demonstrated that early intervention of microbiota using aged chickens can decrease fearfulness, likely through enhanced N-glycan and glutamate metabolism [45], which positively impacts behavior through the gut-brain axis. Conversely, the accumulation of toxic metabolites such as p-cresol sulfate, indole-3-acetic acid, and indoxyl sulfate can impair gut barrier function, allowing toxins to enter the circulatory system [122]. These molecules disrupt the blood–brain barrier and trigger the production of pro-inflammatory cytokines such as interleukin (IL)-1β, IL-6, and tumor necrosis factor alpha, which induce pathological changes in the brain via the Janus kinase-signal transducer and activator of transcription pathway [122].

Serotonin (5-HT) also plays a critical role in regulating chicken behavior. The main source of 5-HT is enterochromaffin cells (EC) in the gastrointestinal epithelium [123]. Certain microorganisms, such as *Clostridium ramosum*, can directly act on EC cells to regulate 5-HT production [124]. Additionally, bacteria including *Lactococcus*, *Lactobacillus*, *Streptococcus*, *Escherichia coli*, and *Klebsiella* synthesize 5-HT using luminal tryptophan [125]. Gut microbes also enhance colonic tryptophan hydroxylase 1 and 5-HT3 receptor mRNA expression through SCFAs, promoting 5-HT production and release [126–128]. While peripheral 5-HT cannot directly cross the blood–brain barrier, gut microbes influence central 5-HT by altering tryptophan metabolism, affecting its availability for brain 5-HT synthesis [125, 129]. Moreover, EC-derived 5-HT stimulates sensory nerves, transmitting discomfort signals to the central nervous system [130]. Notably, MT from the 6_3_ chicken line (a Marek’s disease-resistance chicken line) into newly hatched chicks has been shown to enhance 5-HT metabolism, as indicated by increased tryptophan and 5-HT turnover ratios, resulting in reduced aggressive behavior in recipients [62–64].

Gut microbes also influence behavior through additional mechanisms, such as modulating endocrine signals from enteroendocrine cells or synthesizing neurotransmitters directly [131, 132]. For example, microbes such as *Bacteroides*, *Bifidobacterium*, *Parabacteroides*, and *Escherichia coli* are known to produce the neurotransmitter γ-aminobutyric acid [132]. Although the mechanisms by which MT affects chicken behavior require further investigation, these findings hold promising implications for improving welfare and productivity in the poultry industry.

### Intestinal morphometrics and barrier function

MT indirectly influences poultry growth by modulating intestinal morphology and function, which are crucial for nutrient absorption. The villus-to-crypt (V/C) ratio, an indication of epithelial cell turnover, improves with longer villi and shallower crypts, indicating better nutrient utilization [133]. MT has been shown to increase duodenal villus length while reducing cecal crypt depth [41]. Recipients of MT from Hy-Line Brown hens or high-weight chickens exhibited enhanced jejunal villus height and V/C ratios [48, 117], with similar results observed for ileal morphology [53, 60, 62]. Inoculation of fermented bacteria can also increase the height of the villi in the whole small intestine and the V/C ratio of broiler chickens [80]. In addition, structural improvements, such as increased thickness of jejunal mucosal muscular, muscle, and serosal layers, have been reported in MT-treated chickens [45]. However, MT effects are not always positive. Fluctuations in intestinal morphology have also been reported, including increased jejunal crypt depth, reduced villus height, and lower V/C ratios at early stages, followed by recovery and improvement over time [65]. Similarly, decreased ileal V/C ratios have also been reported in some cases [84].

The intestinal barrier plays a crucial role in preventing harmful substances from entering systemic circulation [134]. This barrier is primarily maintained by intestinal epithelial cells interconnected by tight junction proteins [134]. MT administration has been shown to enhance this barrier by upregulating the expression of key tight junction proteins. For instance, in broilers infected with *Clostridium perfringens*, MT increased the mRNA levels of Mucin (*MUC*) 2B, *MUC13*, and Zonula occludens 1 (*ZO1*) in the jejunum [15]. Similarly, MT in Hy-Line Brown laying hens significantly elevated the expression of *MUC2* and *ZO1* in the ileum [50, 53]. Although a reduction in the expression of Claudin (*CLDN*) 2 in the ileum was reported, MT consistently increased *ZO1* and *CLDN1* levels [80]. Moreover, MT has been observed to enhance *MUC2* expression in both the jejunum and ileum, further strengthening the intestinal barrier [60].

### Immune function

The impact of MT on immune function is extensive. In the following part, we summarize the effects of MT on the development of the chicken immune system, with a focus on the protective role of the mucus layer as the first line of host defense. Additionally, we elucidate the influence of MT on both innate and adaptive immunity in chickens, as well as its effects on the extra-intestinal immune system.

#### The role of MT in immune system development

The chicken immune system comprises central immune organs, peripheral immune organs, and the immune cells they produce. The central immune organs, including the thymus and bursa of Fabricius, provide the initial environment for immune cell development. MT has been shown to promote the development of the thymus and bursa of Fabricius in chickens, mainly by increasing the size of their follicles, medulla, and cortex [51, 53]. The peripheral immune organs of chickens primarily include gut-associated lymphoid tissue (GALT) and the spleen. The development of GALT is significantly influenced by the intestinal microbiota [135]. Chicken cecal tonsils are an essential component of GALT [50]. MT, in conjunction with inulin, has been shown to significantly increase the surface area of the germinal center of chicken cecal tonsils [50]. However, there is currently no direct evidence indicating that MT affects growth of the spleen [51]. Early microbial colonization is essential for maturation of the host’s immune system. Immune immaturity in chickens is associated with increased susceptibility to diseases and higher mortality rates [136, 137]. The immune system of newly hatched chicks is not fully established, making them vulnerable to external factors [31]. This immaturity suggests that application of MT in day-old chicks must be carefully evaluated for safety. Although some studies suggest that MT may induce immune stress, characterized by elevated serum concentrations of IL-1β, IL-4, and IL-6, as well as potential risks of jejunal damage, these findings were observed in chicks at three weeks of age [65, 138]. Further research is needed to assess the safety of MT specifically in newly hatched chicks. Despite limited data on the safety of MT in early life, numerous studies have demonstrated its efficacy in preventing diseases. For instance, MT has been found to enhance chickens' resistance to various pathogens, including *Salmonella* Typhimurium [31, 69, 86], *Salmonella* Enteritidis [61], *Campylobacter jejuni* [46, 69, 82], *Mycoplasma gallisepticum* [55], *Clostridium perfringens* [15], and *Eimeria tenella* [56]. Critical host immune-microbiota interactions occur during early life’s key developmental windows, potentially having long-term effects on various aspects of the immune system [137]. Despite these insights, the mechanisms underlying these interactions in chickens remain poorly understood.

#### MT-induced compartmentalization of the intestine

Under healthy conditions, the host’s immune response to the gut microbiota is strictly localized to the mucosal surface [137]. A stable barrier, consisting of mucus, antimicrobial peptides (AMPs), and sIgA antibodies, separates luminal antigens from the underlying epithelium and serves as the primary defense against microbes and pathogens [51, 139]. The mucus barrier, primarily formed by the highly glycosylated *MUC2*, is essential for this barrier function [137]. MT has been shown to upregulate gene expression of intestinal *MUC2* [15, 50, 53, 60], However, this effect is influenced by the donor microbiota, as some research indicates that MT can exacerbate lipopolysaccharide (LPS)-induced reductions in *MUC2* expression [72]. AMPs and sIgA are also essential components of the intestinal immune barrier, as discussed below.

#### Innate immunity

The microbiota and innate immune system engage in extensive bidirectional communication [137]. AMPs are a critical part of innate defense mechanisms. In poultry, AMPs primarily consist of 14 avian beta-defensins (*AvBD*) and 4 cathelicidins (*CATHs*) [140, 141], with heterophils acting as a significant source of these peptides [142]. Studies have shown that transplanting cecal microbiota from line 7_2_ chickens (a Marek’s disease-susceptible chicken line) increased the heterophil/lymphocyte ratio in the blood [62]. During pathogenic infections, increased AMPs expression aids in pathogen clearance [141]. For instance, chicken *AvBD6* can inhibit the growth of *Escherichia coli*, *Campylobacter jejuni*, *Clostridium perfringens*, *Staphylococcus aureus*, *Saccharomyces cerevisiae*, and *Candida albicans* [143]. Notably, infection with *Clostridium perfringens* led to increased *CATH1* expression in chickens [15]. However, in a necrotic enteritis challenge, MT-treated chickens showed decreased expression of *CATH1* and *AvBD6*. The authors suggested that corticosterone-induced immunosuppression might play a role [15], highlighting the need for further studies on MT's effects on AMPs expression. Future research may explore how MT can manipulate AMPs levels to control selective colonization by pathogens in chickens.

Recognizing microbial pathogens is a key element in initiating innate immune responses, mediated by germline-encoded pattern-recognition receptors (PRRs) such as Toll-like receptors (TLRs). These receptors recognize conserved molecular patterns on pathogens, termed pathogen-associate molecular patterns (PAMPs) [144]. Upon recognizing PAMPs, PRRs activate signaling cascades to trigger protective immune responses [137, 144]. For instance, LPS injection significantly upregulated *TLR7* expression in the chicken ileum, a response mitigated by MT treatment, which reduced LPS-induced *TLR7* elevation [72]. Similarly, transferring microbiota from ammonia-exposed broilers to healthy broilers has been shown to cause gut damage and increase ileal *TLR4* expression in the recipients [145]. While most PRRs research focuses on infection contexts, these findings also suggest that PRRs play an essential role in sensing pathogens [146]. Importantly, PAMPs are not exclusive to pathogens; they are abundantly produced by resident microbiota during normal colonization. Interestingly, PRR activation does not always lead to inflammation; it can also enhance host development and immunity [146]. For example, in mice, the gut symbiont *Bacteroides fragilis* was shown to produce polysaccharide A, which promoted its survival on mucosal surfaces by activating TLR2 [147]. Similarly, MT in chickens has been shown to reduce *TLR4* expression in the rectum [51] or jejunum [48]. Despite these insights, how TLRs differentiate between symbiotic microbes and pathogens to prevent unnecessary inflammatory responses remains unclear, warranting further exploration. Other PRRs, including membrane-bound C-type lectin receptors, cytosolic NOD-like receptors, and RIG-I-like receptors, are also involved in PAMPs recognition and innate immune control [144]. However, the relationship between MT and PRRs in chickens remains poorly understood, warranting additional investigation.

Dendritic cells (DCs) and macrophages are critical innate immune effector cells. MT has been shown to significantly increase ileal DCs in chickens [31]. The recruitment of chicken macrophages is heavily reliant on the presence of a microbiota [148]. MT strongly promotes macrophage development and polarization, as evidenced by upregulation of macrophage polarization-related cytokine genes, such as CC ligand 17 and IL-8 following MT treatment [31]. Additionally, early exposure to adult microbiota has been linked to increased activation of innate immune cells, such as natural killer cells [85].

#### Adaptive immunity

The thymus and bursa of Fabricius are essential organs for the development of T cells and B cells, respectively. In chickens, the critical window for immune cell development occurs between 14 and 28 days of age [31]. During this period, immune cells populate the peripheral immune system, where they undergo further maturation and differentiation [149]. T cells are categorized into CD4^+^ or CD8^+^ subsets based on the expression of their co-receptors. Early exposure to mature microbiota has been shown to increase the proportion of CD4^+^ T cells in the ileum [31, 50].

The relative abundance of CD8αα^+^ T cells in the gut is maintained within a balanced range, contributing to immune homeostasis [55]. However, MT with microbiota derived from *Mycoplasma gallisepticum*-infected chickens has been associated with an increased CD4^+^/CD8^+^ T cell ratio in the recipients’ blood [55]. Additionally, MT has been reported to increase the relative abundance of CD8αα^+^ T cells in the gut, potentially altering local immune regulation [85]. Among CD4^+^ T cells, functional subtypes include regulatory T cells (Tregs) and helper T cells (Th), which normally exist in a dynamic balance essential for immune regulation. Notably, Ma et al. [42] observed that MT modulated Th17/Treg-related transcription and cytokine profiles in the jejunum. This modulation helps protect the gut from inflammation and promotes intestinal homeostasis. However, MT with microbiota derived from infected chickens can disrupt the Th1/Th2 balance [55].

Gut microbiota dysbiosis has been shown to impact the maturation and differentiation of B cells. For instance, administering chickens with antibiotics significantly reduced the proportion of total B cells in the peripheral blood at 14 days of age [31]. Studies suggest that early exposure to mature microbiota promotes the abundance of intestinal B cells, indicating that MT can support B cell development [31, 60]. This finding was corroborated by Song et al. [50], who demonstrated that MT combined with inulin supplementation enhanced the expression of B cell markers (*BAFFR*, *PAX5*, and *Bu-1*) in the cecal tonsil.

Germ-free animal models offer a valuable tool for exploring the causal relationship between symbiotic microbiota and adaptive immunity. In germ-free chicken models, transcription levels of immune factors such as IL-4, IL-8, IL-10, and interferon-gamma in the ileum were significantly reduced. However, following MT, transcription levels of these immune factors increased, accompanied by higher proportions of CD3^+^, CD4^+^ T cells, and B lymphocytes [60]. sIgA is considered a primary immune barrier for maintaining symbiotic microbiota homeostasis and is primarily produced by B cells, with its production being regulated by T cell-derived cytokines [137]. MT has been consistently reported to enhance sIgA expression in the gut, as demonstrated across multiple studies [31, 60, 62, 65].

#### MT on extra-intestinal organ immunity

While much of the research has focused on the relationship between the gut microbiota and intestinal immunity, the interactions between gut microbiota and extra-intestinal organs immunity have garnered increasing attention. One such interaction is the gut-lung axis, which highlights the critical interplay between the gut microbiota and pulmonary health. *Mycoplasma gallisepticum* infection is associated with pulmonary inflammation and damage [55]. MT from *Mycoplasma gallisepticum* infected chickens to recipients resulted in pulmonary inflammation, accompanied by compromised intestinal mucosal barriers and reduced host defenses against *Escherichia coli* [55].

The gut-liver axis highlights the direct anatomical connection between the gastrointestinal tract and the liver via the portal vein and bile duct systems, which exposes the liver to microbial products derived from the gut [150]. MT has been shown to mitigate liver inflammatory lesions caused by *Salmonella* Enteritidis (SE) infection in chicks, as evidenced by no typical SE colonies being detected on a *Salmonella*-specific screening medium, suggesting that MT was capable of inhibiting SE colonization in the liver [61]. Symbiotic gut bacteria and their products can translocate from the intestinal lumen to the liver under certain conditions, potentially influencing hepatic immune responses [137]. The authors propose that the inhibitory effect on SE may be linked to SCFAs. In SE-infected groups, MT restored intestinal acetate levels to those of normal controls and promoted increased butyrate levels in the gut [61].

The gut-brain axis underscores the influence of the gut microbiota on the brain's immune system and behavior through microbial metabolites (see the Behavior section). While significant progress has been made in understanding the interactions between the gut microbiota and extra-intestinal organs, large knowledge gaps remain, particularly in the context of MT and its impact on extra-intestinal organ immunity in chickens. Further research is needed to elucidate these complex relationships.

### Bone and fat metabolism

Broiler chickens grow rapidly, but when bone development cannot keep up with increasing body weight, health issues often arise [151]. In contrast, laying hens experience continuous deposition of medullary bone in the bone marrow cavity over time, leading to weakened bone metabolism with age [152]. Research indicates that transferring microbiota from hens to chicks can significantly increase the tibia length of broilers [117]. Additionally, transplanting the microbiota of healthy chickens into chickens suffering from tibia dyschondroplasia can restore bone development. This mechanism is related to the gut microbiota’s role in regulating hyperglycemia and bone metabolism [153]. Moreover, the gut microbiota can enhance phosphorus absorption by upregulating intestinal type IIb sodium-dependent phosphate cotransporter mRNA expression [154], or by creating an acidic environment in the gut through SCFAs, which increases the solubility of calcium and other minerals [155]. However, the exact mechanisms of MT on bone metabolism remain unclear.

Fat metabolism is a complex biochemical process that is significantly influenced by the gut microbiota in chickens. Transferring microbiota from high-weight adult chickens to newborn chickens improved liver fat metabolism by remodeling the gut microbiota composition, as evidenced by the successful transfer of *Sphingomonas* and *Microbacterium*, which are key features of high-weight chickens [71]. Transferring the microbiota from adult chickens with low abdominal fat deposits to newborn chickens significantly decreased the expression of genes related to fat synthesis while increasing the expression of genes associated with fat breakdown in abdominal fat and the liver [54]. Conversely, transferring the microbiota from chickens with high abdominal fat deposits reduces the variability in abdominal fat traits and promotes fatty acid elongation [66]. Folic acid supplementation also reduces fat deposition. Notably, transferring the microbiota from chickens supplemented with folic acid to recipient chickens results in reduced fat accumulation, demonstrating a causal relationship between MT and fat metabolism [68]. Additionally, MT using local breeds of chicken (Beijing-You broilers) increased abdominal fat content, reduced muscle drip loss, and increased biceps muscle fiber diameter [47].

## Future perspectives on the application of MT

At present, the application of MT in chickens is hindered by the absence of standardized procedures. While standardized MT protocols have been developed for pigs [156], no such guidelines are available for poultry. Thus, there is a critical need to establish standardized MT protocols for chickens in the future. One major challenge is the variability in dilution ratios, ranging from 1:1 to 1:100. Although dilution ratios of 1:6 and 1:10 are commonly used (Tables 1 and 2), there is no consensus on the optimal microbiota concentration. The microbial load in the chicken small intestine is approximately 10^5^ CFU/g digesta [18], while the cecum harbors around 10^10^ CFU/g digesta [18], suggesting that inocula for MT likely fall within this range. Studies have used as few as 10^4^ CFU for gavage in chicks, with little impact on phenotypic traits [40]. However, there is no direct evidence establishing a relationship between CFU levels and MT success rates. Future research should focus on determining the optimal microbiota concentration to maximize MT efficacy in chicken.

In some studies, brown sugar [44], cysteine [37], or glucose [45] is added to fecal suspensions before gavaging chicks. Most studies incorporate 10% glycerol to prevent microbial damage caused by ice crystal formation during freezing. The choice of diluent also varies, with saline and phosphate-buffered saline being the most commonly used, though their compositions differ between studies. In addition, the processing environment significantly impacts bacterial viability. Studies have shown that under ambient air homogenization conditions, bacterial viability is around 19%, the abundance of important commensal taxa decreases 12-fold, and butyrate and acetate production slurry is significantly reduced. In contrast, strictly anaerobic processing of feces retains about 50% bacterial viability [36].

Donor screening is a critical step prior to MT application. For instance, autopsies of deceased recipient chickens showed that they had *Eimeria* infections and hemorrhagic typhlitis, which attributed to the inoculation fluid containing oocysts from five different *Eimeria* species [73]. Similarly, chickens inoculated with cecal contents from donor hens exhibited higher counts of representative *Campylobacter* species than those found in the donor’s microbiota [37]. Although the presence of pathogen in inocula does not always correlate with clinical symptoms, as it depends on pathogen concentrations [73], these findings underscore the potential risk of pathogen transfer from donor to recipient, highlighting the need for rigorous screening protocols to ensure MT safety.

Moreover, the implementation methods of MT need to be given special consideration for its large-scale application in the future. Currently, most MT administration methods are through a gavage, but this would seem difficult to use on a large scale in poultry production. Few experiments have shown that the purpose can be achieved through drinking water or repeated use of bedding. But more experiments are needed to prove its effectiveness. In addition, it is recommended to use it in combination with prebiotics in practice given their aforementioned benefits on the establishment of an ecological niche.

Establishing a stool bank is considered a reasonable approach for applying MT in human medicine. Hu et al. [156] also emphasized the necessity of stool banks for MT applications in pigs. However, this approach may not be suitable for broilers due to their short growth cycle, as maintaining a stool bank would increase costs. In contrast, for laying hens with longer lifespans, the use of a stool bank remains worth considering, especially since studies have shown that MT can improve egg production performance. Rather than focusing on stool banks, identifying core microbiota through MT and establishing a probiotic bank may be more important. For instance, Zhang et al. [57] used targeted culture omics to isolate 6 species of *Lactobacillus* and *Bacillus* from recipients and confirmed their individual effects on chicken growth performance, providing valuable insights for probiotic selection. Furthermore, MT revealed significant increases in *Methanobrevibacter* and *Mucispirillum schaedleri* abundance in recipients, with correlation analyses linking these changes to enhanced fat deposition [66]. Certain Bacteroidetes members in the recipient group, such as *Bacteroides*, *Rikenellaceae_RC9_gut_group*, and *Prevotellaceae_UCG_001*, were strongly associated with resistance to *Salmonella* Enteritidis infection [61]. Thus, MT enables identification of key microbiota influencing recipient phenotypes, providing the foundation for targeted probiotic screening.

## Conclusions

In conclusion, while MT offers promising benefits in chickens, such as improved performance, behavior, gut health, immunity, and various metabolic processes, its application comes with significant challenges, such as those discussed in this paper regarding donor selection, material processing, and administration methods. Despite these challenges, MT holds great potential for application to chickens. Moreover, MT is not only a tool for improving host health but also serves as a valuable model for fundamental research. This is particularly important in microbiota studies, where the causal relationship between microbial changes and host physiological responses is often unclear. To maximize the advantages of MT, future research should focus on standardizing MT protocols, optimizing delivery methods, and, most importantly, leveraging MT as an effective research model to better understand microbiota-host interactions.

## Data Availability

Not applicable.

## Abbreviations

5-HT: Serotonin
AMPs: Antimicrobial peptides
*AvBD*: Avian beta-defensins
*CATHs*: Cathelicidins
CDI: *Clostridioides difficile* Infection
CFU: Colony-forming units
*CLDN*: Claudin
DCs: Dendritic cells
EC: Enterochromaffin cells
FMT: Fecal microbiota transplantation
FP: Feather pecking
GALT: Gut-associated lymphoid tissue
GIT: Gastrointestinal tract
HFP: High feather pecking
IL: Interleukin
LFP: Low feather pecking
LPS: Lipopolysaccharide
MT: Microbiota transplantation
MUC: Mucin
PAMPs: Pathogen-associated molecular patterns
PRRs: Pattern-recognition receptors
SCFAs: Short-chain fatty acids
sIgA: Secretory immunoglobulin A
Th: Helper T cells
TLRs: Toll-like receptors
Tregs: Regulatory T cells
V/C: Villus-to-crypt
*ZO1*: Zonula occludens 1
